# The role of the right inferior frontal gyrus in the pathogenesis of post-stroke psychosis

**DOI:** 10.1007/s00415-014-7242-x

**Published:** 2014-01-22

**Authors:** Michael J. Devine, Paul Bentley, Brynmor Jones, Gary Hotton, Richard J. Greenwood, I. Harri Jenkins, Eileen M. Joyce, Paresh A. Malhotra

**Affiliations:** 1 Division of Brain Sciences, Imperial College London, 10 E Charing Cross Campus, London, W6 8RP UK; 2 Imperial College Healthcare NHS Trust, Charing Cross Hospital, Fulham Palace Road, London, W6 8RF UK; 3 UCL Institute of Neurology, Queen Square, London, WC1N 3BG UK

**Keywords:** Stroke, Psychosis, Delusions, Inferior frontal gyrus

## Abstract

Psychotic symptoms have previously been reported following right hemisphere brain injury. We sought to identify the specific neuroanatomical basis of delusions following stroke by studying a series of patients with post-stroke psychosis. Lesion overlap analysis was conducted on three individuals with delusions following right hemisphere stroke. These cases were compared with a control group of patients with similar anatomical damage. The main outcome measures were presence of delusions and presence of behavioural susceptibility. The right inferior frontal gyrus and underlying white matter, including the superior longitudinal fasciculus and anterior corona radiata, were involved in all three cases. All three had a preexisting untreated psychiatric disorder. In contrast, only one of nine control cases with equivalent lesions had evidence of previous psychiatric disorder (*p* = 0.0182, Fisher’s exact test), and this was being treated at the time of stroke. We provide clinical evidence from patients with structural brain lesions implicating damage to the right inferior frontal lobe in the generation of persistent psychosis following stroke. We suggest that preexisting psychiatric disease provided a behavioural susceptibility to develop delusions in these individuals.

**Electronic supplementary material:**

The online version of this article (doi:10.1007/s00415-014-7242-x) contains supplementary material, which is available to authorized users.

## Introduction

Psychosis is a syndrome involving one or more of delusional beliefs, hallucinations, and thought disorder. Psychotic symptoms have been consistently noted in the context of neurodegenerative disease [15] and focal cerebral lesions, particularly those affecting the right cerebral hemisphere [4, 7, 8, 14]. Post-stroke delusions have often been reported in the context of abnormal bodily perception such as somatoparaphrenia or anosognosia [8], but behaviour more characteristic of psychotic disorders has also been described, albeit infrequently [7, 14].

To date, there has been no clear association of a specific neuroanatomical locus with psychotic symptoms, although delusions have been reported following strokes affecting the right frontal, parietal, and temporal lobes, as well as subcortical structures [7, 10]. To our knowledge, there has been no use of lesion overlap analysis with a standardised MRI-based template to identify a common region of damage in a group of patients with persistent symptoms. Furthermore, no previous reports have indicated why only a small proportion of right hemisphere strokes cause delusions, although it has been suggested that pre-existing cerebral atrophy may play a role [8, 14].

Here we describe three individuals with a history of psychiatric disorder, but without previous hallucinations or delusions, who had right hemisphere strokes causing persistent delusions unrelated to body awareness or anosognosia. We conducted a lesion overlap analysis, in order to identify specific brain loci involved in the generation of psychosis.

## Patients and methods

**Fig. 1:**
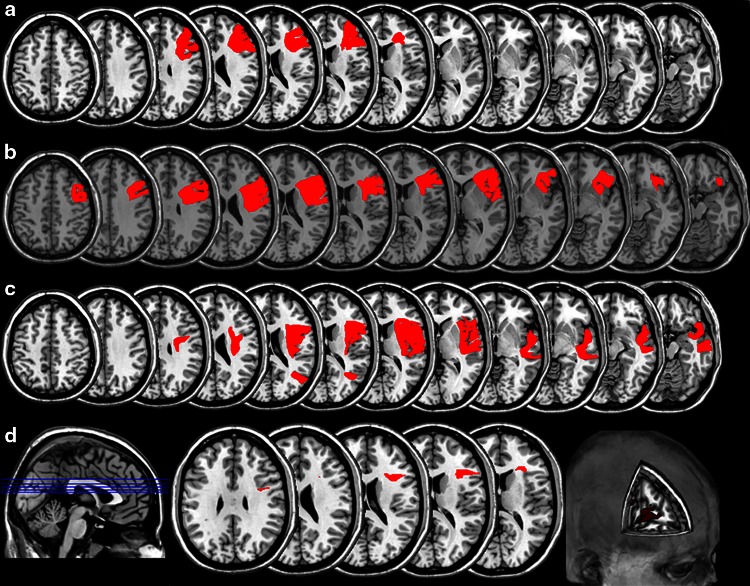
**a–c** Lesions. Case 1 suffered from a haemorrhagic stroke, whereas Cases 2 and 3 had ischaemic lesions. **d** Lesion Overlap. There was lesion overlap at five slice levels (displayed in *blue* on sagittal image). The overlap region (displayed in *red* on axial and cutout images) was damaged in all three cases (Centre: MNI coordinates 46,23,16)

Please see Online Supplement for Individual Case Histories. All three patients were noted by neighbours and/or family to have been behaving abnormally for the preceding 24–72 h. Neurological examination on admission excluded significant hemiparesis, sensory loss, or visual deficit. All were right hand-dominant. Imaging studies revealed right hemisphere stroke in all cases (Fig. 1a–c).

All three patients had developed prominent persecutory delusions. They also experienced at least one episode of auditory hallucinosis during their admission, and two patients (Cases 1 and 2) manifested delusional misidentification. Organic psychosis secondary to hemispheric stroke was diagnosed in each case and treatment with antipsychotics was initiated, but all three continued to have persistent delusions at least 6 weeks post-admission, requiring transfer to long-term mental health units. All three patients had pre-existing mental health problems: depression (untreated), anxiety, or alcohol abuse. None had a history of psychosis. Neuropsychometric profiling revealed frontal executive dysfunction in all three cases.

Patient scans were reviewed by a Neuroradiologist (BJ). Visual inspection of these scans found minimal background regional volume loss and minimal evidence of previous cerebrovascular disease. Lesions were mapped onto twelve axial slices of a T1-weighted anatomical template (ch2 MNI) using MRIcro software (http://www.cabiatl.com/mricro/) using direct visualisation of their clinical scans [one CT (Case 1) and two MRI scans] (see Fig. 1a–c). Overlap analysis was carried out in MRIcro including use of an ICBM-based white matter atlas [11].

In order to try to identify potential factors contributing to the development of psychosis, these patients were compared to control patients with damage to similar regions, identified from a lesion database of 430 patients with stroke. Statistics were calculated using GraphPad Prism software.

## Results

Lesion overlap analysis demonstrated that right frontal cortex and underlying white matter damage was common to all cases (Fig. 1d). The main cortical region involved was the inferior frontal gyrus (IFG) (center of region: MNI coordinates 46,23,16). Further analysis of the overlap volume of interest (VOI) using the ICBM DTI81 atlas [11] showed that damaged white matter structures comprised superior longitudinal fasciculus (MNI coordinates 33,0,30) and anterior corona radiata.

Queries in our lesion database of 430 individuals for patients with damage to MNI coordinates 46,23,16 identified nine patients (please see Online Supplement for Supplementary Table I). None of these patients manifested signs of psychosis on admission, and examination of their records revealed no history of preceding psychiatric disorder apart from one individual (Control Case 3) who was receiving antidepressant therapy on admission for mild depression. Cases and controls were compared with respect to presence or absence of preexisting psychiatric disorder, and found to be significantly different (*p* = 0.0182, Fisher’s exact test).

## Discussion

Post-stroke psychosis is uncommon and unexplained. Only 15 of 360 stroke patients suffered from delusions in one large case series [7]. Such delusions usually involve those of bodily perception, and are generally transitory [7, 8, 14]. Previous studies have not used lesion overlap analysis to determine a common area of damage in patients with psychosis following stroke. Here we demonstrate the most discrete area of involvement to date: right IFG and underlying white matter was common to all three patients with persistent post-stroke psychosis. Frontal lobe involvement has been documented previously, but only in the context of widespread damage to other areas [7, 8, 14]. Our findings are consistent with observations of patients with degenerative disease, where psychotic symptoms are associated with right-sided atrophy [13]. Delusional misidentification, manifested by two of our patients, is also associated with hypoperfusion of the inferior frontal gyri in patients with degenerative disease [12].

Functional imaging in psychotic patients has demonstrated an increase in right inferior frontal activity in association with auditory hallucinations [17]. Impaired functional connectivity, especially of right inferior frontal lobe, appears to be a key factor in the pathogenesis of psychosis [1, 18] and superior longitudinal fasciculus involvement may be particularly important [6, 16]. As a corollary, our findings suggest that focal lesions to right IFG and neighbouring white matter might cause impaired anatomical connectivity in the three patients described, possibly via potentiating symptom-associated neural overactivity within the disconnected (and thus newly disinhibited) cortex. However, because all three patients had damage to right IFG as well as associated white matter, we are unable to differentiate which of these relates more closely to the generation of psychosis, or if both are required.

Why is post-stroke psychosis so rare? Structural and functional imaging studies of delusions have suggested that more than one focus of abnormality has to be present for delusional ideas to become severe enough to clinically manifest. A recent meta-analysis examined the neuroanatomical correlates of vulnerability to psychosis by comparing structural MRI findings between subjects at enhanced risk of developing psychosis and controls [5]. Whilst a number of areas differed between these two groups, there was a specific gray matter volume reduction in right IFG only in high risk subjects who subsequently developed a psychotic episode. Functional imaging studies of schizophrenia have found that prediction error signalling (denoting the mismatch between what is expected and what is experienced) is attenuated in right frontal areas in delusional patients, and the degree of attenuation correlates with the severity of their unusual thoughts [3]. These authors propose that inappropriate prediction error signalling associated with abnormal right frontal cortical function leads to delusions via inaccurate representations of the environment. More recently they have found that healthy people with non-clinical schizotypal beliefs have the same abnormalities in prediction error signalling in right IFG as psychotic patients, demonstrating that premorbid vulnerabilities exist in healthy people [2]. These structural and functional imaging studies support the view that psychotic symptoms exist as part of a continuum, with schizotypy occurring as an attenuated form of clinical psychosis [9]. Furthermore they also suggest that multiple ‘hits’ are required for psychosis to manifest clinically, and that right IFG dysfunction is a critical factor. In our study, we suggest that right IFG damage due to stroke was the second hit which triggered psychosis, the first hit being their untreated mental health problems. Although cerebral atrophy has been highlighted as a potential factor in generating post-stroke psychotic symptoms [8, 14], none of our patients had atrophy and, given the high prevalence of moderate atrophy amongst stroke patients, a much higher incidence of similar symptoms might be expected if this were contributory. We suggest that these three individuals may have had a behavioral susceptibility to psychosis, related to their pre-existing mental health problems. This would explain the relatively low incidence of post-stroke psychosis, since the large population of patients who suffer from stroke in the same vascular territory, but have no significant psychiatric history, do not develop similar symptoms.

Our findings are preliminary; the number of cases is small, due to the rarity of the condition, and premorbid schizotypy was not looked for specifically. In future large scale studies of post-stroke psychosis, it would be informative to screen for schizotypal personality as well as premorbid psychiatric disease. Ours and previous studies would predict that individuals with schizotypal features would be at a substantially higher risk of developing post-stroke psychosis if right IFG is involved. Additional cases would also be needed to determine other risk factors, behavioural or structural, that predispose to psychosis following stroke.

To summarise, our study provides the most precise lesion data to date corroborating existing structural and functional studies that right IFG damage is a critical component in the development of clinical psychosis in at-risk individuals. Moreover, from the practical point of view, in a clinical setting it might be worth making the extra effort to clarify an individual’s previous psychiatric history when they appear to be manifesting delusions in the context of hemispheric stroke.

## Electronic supplementary material

Below is the link to the electronic supplementary material.

Supplementary material 1 (DOCX 26 kb)
