# Supplementary material for: The role of the right inferior frontal gyrus in the pathogenesis of post-stroke psychosis
Source: J Neurol. 2014 Jan 22;261(3):600–3. doi: 10.1007/s00415-014-7242-x (PMC3948509; doi:10.1007/s00415-014-7242-x)
Supplement: Supplementary file 1 — Supplementary material 1 (DOCX 26 kb) [file 415_2014_7242_MOESM1_ESM.docx]

**ONLINE SUPPLEMENT**

**Title:** The role of the right inferior frontal gyrus in the pathogenesis of post-stroke psychosis

**Journal Title**: Journal of Neurology

**Authors:** Michael J. Devine^1,2^, Paul Bentley^1,2^, Brynmor Jones^2^, Gary Hotton^3^, Richard J. Greenwood^3^, I. Harri Jenkins^2^, Eileen M. Joyce^3^, Paresh A. Malhotra^1,2^

**Affiliations:**

^1^Division of Brain Sciences, Imperial College London, 10 E Charing Cross Campus, London W6 8RP, UK

^2^Imperial College Hospitals NHS Trust, Charing Cross Hospital, Fulham Palace Road, London W6 8RF, UK

^3^UCL Institute of Neurology, Queen Square, London WC1N 3BG, UK

**Corresponding author:**

Paresh A. Malhotra

Centre for Neuroscience, Imperial College London, 10 E Charing Cross Campus, London W6 8RP, UK

Tel +44 203 311 7286, Fax +44 203 311 7286, E-mail: [p.malhotra@imperial.ac.uk](mailto:p.malhotra@imperial.ac.uk)

**Individual Case Histories**

The three patients presented between 2006 and 2009. Cases 1 and 2 were admitted to Imperial College Healthcare NHS Trust and Case 3 was admitted to the National Hospital for Neurology and Neurosurgery (both in London, UK).

**Case 1:** A 78 year old right-handed man presented with three days of confusion. His family noted that he had become vague, slow to answer questions and was behaving out of character. He reported difficulty with short term memory, transferring thoughts to speech, and orientation in time. The day prior to presentation he had developed slurred speech, became unsteady on his feet, and experienced episodic left leg weakness on descending stairs. On direct enquiry, he admitted to a two day history of persecutory delusions about “them”, “people are keeping me here”, “sexual things … physical abuse happening to my daughters” and had alternating beliefs that his deceased wife was “non-existent” or alive.

He was a retired pharmacist, who lived independently. He had experienced a possible transient ischaemic attack two and a half years previously. He was hypertensive and had hypercholesterolaemia. He had stopped smoking 20 years before and did not drink alcohol. Following his wife’s death from lung cancer two years previously, he had become depressed. Over this time he had become withdrawn, disinterested in his appearance, with poor appetite and had lost one stone in weight. He had turned down anti-depressant therapy and counselling.

At presentation, he was poorly cooperative, suspicious and disinhibited. On examination his gait was noted to be unsteady and slow, and he was mildly dysarthric. Limb examination was unremarkable except for absent ankle jerks. Plantar responses were flexor. He also had upper limb apraxia and mild left-sided neglect. CT brain demonstrated a subacute right frontal haematoma and he was admitted to the stroke unit.

During his admission he persistently thought that female carers were his daughters (Fregoli syndrome). In addition, he believed that the hospital was inserting thoughts into his mind. Repetitive face washing was observed. One week into admission he became agitated, threw water over a nurse and smashed a window. This prompted a psychiatric review where no perceptual abnormalities were detected, and the impression was of an organic psychosis in an at-risk individual (in the context of a moderate depressive episode due to an abnormal/prolonged grief reaction). The neuropsychologist and rehabilitation therapists documented that, at times, he said that he could hear his wife’s voice.

Formal neuropsychological testing showed verbal fluency to be reduced with perseveration after category shift. Minnesota cognitive testing found severe difficulties with attention span, digit span, sequencing, recall of words and actions, complex problem solving and mental flexibility. There were no difficulties with remote and immediate memory, auditory comprehension, object recognition, and following of verbal or written directions. An EEG showed diffuse slowing with no features of encephalitis or seizures.

Quetiapine was started on hospital day nine. Insight improved over the following two weeks such that he was able to understand that his wife was deceased. Doses were tapered one month after commencement because he appeared cognitively slower, but five days later he smashed a window, because he believed that terrorists were attacking people on the ward. He also lashed out at a member of staff looking after him, and smashed a fire alarm because he believed people were carrying dead bodies around the ward and that an abortion was being carried out. Quetiapine was switched to haloperidol without benefit. He was subsequently transferred to a mental health unit for ongoing management six weeks post admission.

**Case 2:** A 64 year old right-handed man presented with 24 hours of apparent confusion. The police were alerted when he was found trying to break into a neighbour’s room, believing it to be his own. His flat was found to be in a chaotic state with food on the floor, and an electric kettle had melted on the cooker. He had visited his family general practitioner three weeks previously for a chest infection and the doctor recalled that he was rational and lucid at the time.

He had been living independently. Previous medical history included chronic obstructive pulmonary disease with recurrent infective exacerbations. He stopped smoking 20 years previously. Medications were inhalers only. He was alcohol-dependent until 1984 when he stopped drinking completely.

At presentation, he was unkempt, sleepy and uncooperative. He was noted to be disoriented in time, place and person. He was also noted to be stressed, agitated and episodically suspicious. Although he could hold a conversation, he seemed to be perseverating. Neurological examination showed no major abnormalities apart from mild dysarthria. Cranial nerves and limb examination were unremarkable. CT brain demonstrated a subacute right frontal middle cerebral artery infarct and he was admitted to the stroke unit.

Neuropsychological evaluation showed impaired digit span, word finding, object recognition, fluency, reasoning and visuospatial skills. He was able to read and there was no evidence of spatial neglect. Infective, metabolic and toxic causes for his confusion were excluded. EEG demonstrated regional cortical dysfunction post-infarct, with no features suggestive of encephalitis or seizures.

He was noted to be experiencing auditory hallucinations of his brother’s voice, and he believed that his brother was in the psychology department. He had marked visuospatial impairment and executive dysfunction, characterised by perseveration and difficulty shifting cognitive set. His delusions interfered with rehabilitation and he persistently believed that medical students were his brother and sister-in-law (Fregoli syndrome).

Quetiapine was commenced one month into admission. Ten days later he became increasingly aggressive and hit two of the ward staff. He thought that there had been a shooting on the roof of the hospital. Quetiapine was switched to haloperidol, but he remained delusional, listening to a wall to hear what was being said about him. He thought that he was being poisoned and refused aspirin on one occasion. He was transferred for ongoing care ten weeks post-admission.

**Case 3:** A 62 year old right-handed woman presented having been confined to bed for three days, with unsteadiness on her feet when she mobilised and slurred speech.

She had been seen by her general practitioner for an anxiety state 12 years previously, but had no other significant past medical history apart from hypercholesterolaemia.

At presentation, she was uncooperative with interventions, tangential in speech, and intermittently agitated. Facial droop and increased tone, minimal pyramidal weakness and brisk reflexes were all noted on the left side. CT head demonstrated a right frontal infarction and she was admitted to the stroke unit.

Initial neuropsychological assessment demonstrated poor anterior functioning, with relative sparing of visuo-perceptual and nominal functions.

On one occasion she absconded, and managed to reach her home but was then brought back to the ward by police, with some resistance. She was found to be more overtly anxious on her return, with persecutory ideation and thought disordered speech. She spoke of Jesus and the devil, and tried to cover windows with newspaper to stop people watching her. She was transferred to the neuropsychiatric unit for further management. On transfer there, she was noted to be unkempt, needing encouragement with self-care, guarded, reluctant to speak about her background history and she felt that staff were sons of devils and were spying on her. On one occasion she stated that she could hear a child outside calling for help, whom she tried to rescue.

Olanzapine was commenced two weeks after neuropsychiatric admission and increased to 15mg per day over the following week, without major impact upon psychotic features. She remained disruptive and at times aggressive on the ward, without insight, and was transferred to a mental health unit for ongoing management six weeks following admission.

**Supplementary Table I**

| **Subject** | **Age** | **Presentation** | **Past Medical History** | **Medications prior to admission** |
| --- | --- | --- | --- | --- |
| **Control Case 1** | 55 | Left hemiparesis, visual extinction | Hypertension, previous right hemisphere stroke | Amlodipine, Ramipril, Aspirin |
| **Control Case 2** | 82 | Left hemiparesis, visual neglect | Prostatic cancer | Nifedipine, Temazepam |
| **Control Case 3** | 49 | Left hemiparesis | Obesity, depression (treated) | Citalopram |
| **Control Case 4** | 65 | Left arm weakness | Previous right occipital infarct | Nil |
| **Control Case 5** | 26 | Left facial weakness | Nil | Nil |
| **Control Case 6** | 42 | Left hemiparesis | Migraine | Sumatriptan as required |
| **Control Case 7** | 77 | Left hemiparesis | Hypertension, Chronic back pain | Nil |
| **Control Case 8** | 67 | Left hemiparesis | Hypertension, gout, atrial fibrillation | Warfarin |
| **Control Case 9** | 85 | Left hemiparesis | Atrial fibrillation, Alzheimer’s disease | Donepezil, Lansoprazole |
